# The usefulness of the McGrath MAC laryngoscope in comparison with Airwayscope and Macintosh laryngoscope during routine nasotracheal intubation: a randomaized controlled trial

**DOI:** 10.1186/s12871-017-0451-y

**Published:** 2017-12-01

**Authors:** Aiji Sato (Boku), Kazuya Sobue, Eisuke Kako, Naoko Tachi, Yoko Okumura, Mayuko Kanazawa, Mayumi Hashimoto, Jun Harada

**Affiliations:** 1 Department of Anesthesiology, Aichi Gakuin University School of Dentistry, 2-11 Suemori-dori, Chikusaku, Nagoya, 464-8651 Japan; 2 Department of Anesthesiology and Intensive Care Medicine, Nagoya City University Graduate School of Medical Sciences, 1 Kawasumi, Mizuho-cho, Mizuho-ku, Nagoya, 467-8601 Japan

**Keywords:** McGrath, Airway scope, Nasotracheal intubation, Intubation time, Cormack Lehane grade

## Abstract

**Background:**

McGrath MAC video laryngoscope offers excellent laryngosopic views and increases the success rate of orotracheal intubation in some cases. The aim of this study was to determine the usefulness of McGrath MAC for routine nasotracheal intubation by comparing McGrath MAC with Airway scope and Macintosh laryngoscope.

**Methods:**

A total of 60 adult patients with ASA physical status class 1 or 2, aged 20–70 years were enrolled in this study. Patients were scheduled for elective oral surgery under general anesthesia with nasotracheal intubation. Exclusion criteria included lack of consent and expected difficult airway. Patients were randomly allocated to three groups: McGrath MAC (*n* = 20), Airway scope (*n* = 20), and Macintosh laryngoscope (*n* = 20). After induction, nasotracheal intubation was performed by six expert anesthesiologists with more than 6 years of experience.

**Results:**

There were no significant differences in preoperative airway assessment among the three groups. Successful tracheal intubation time was 26.8 ± 5.7 (mean ± standard deviation) s for McGrath MAC, 36.4 ± 11.0 s for Airway scope, and 36.5 ± 8.9 s for Macintosh laryngoscope groups. The time for successful tracheal intubation for McGrath MAC group was significantly shorter than that for Airway scope and Macintosh laryngoscope (*p < 0.01*). McGrath MAC significantly improved the Cormack Lehane grade for nasotracheal intubation compared with Macintosh laryngoscope (*p < 0.05*).

**Conclusion:**

McGrath MAC significantly facilitates routine nasotracheal intubation compared with Airwayscope and Macintosh laryngoscope by shortening the tracheal intubation time and improving the Cormack Lehane grade.

**Trial registration:**

UMINCTR Registration number UMIN000023506. Registered 5 Aug 2016.

## Background

Recently, various types of video laryngoscopes have been introduced for securing the airway during general anesthesia. McGrath® MAC video laryngoscope (hereafter referred to as “McG,” Covidien, Tokyo) was released in 2012. Its structure is similar to that of a conventional Macintosh laryngoscope (hereafter referred to as “ML”); therefore, tracheal intubation is possible under direct vision using only the display mounted on top of McG handle [1]. McG has been used for orotracheal intubation in both normal patients and patients for whom intubation was expected to be difficult, and has been reported to provide improved visibility of the glottis during tracheal intubation as well as an increased tracheal intubation success rate [2, 3], although Ng et al. reported that the C-MAC vedeolaryngoscope allowed a quicker intubation time and attempts compared with the McGrath videolaryngoscope when used in patients with Mallampati grade of > 3 [4]. On the other hand, Airway Scope® AWS-S100 (hereafter referred to as “AWS,” Pentax, Tokyo) is a video laryngoscope released in 2010. The Intlock (disposable single-use blade) bears a J-shape, which is anatomically designed to suit the pharynx and larynx. It is possible to view the glottis with AWS without the need for direct laryngoscopy or adopting the sniffing position [5]. Even doctors and dentists who do not usually perform tracheal intubation in the course of their daily clinical practice can reportedly perform rapid tracheal intubation with orotracheal intubation using AWS [6], thereby firmly establishing the usefulness of AWS in orotracheal intubation. However, there are published recommendations to use AWS for nasotracheal intubation (routine nasotracheal intubation for 103 patients in Maxillofacial hospital [7], meta-analysis of randomized controlled trial [8], and case series for nasotracheal intubation in patients with difficult airways [9]. Also there is a report which describes that the AWS offers better intubation conditions than the ML during nasotracheal intubatin [10]. On the other hand, nasotracheal intubation is reportedly easier with McG than with ML [11]. The authors were unable to locate any reports comparing the outcomes of nasotracheal intubation with the two systems.

This study determined the usefulness of McG in comparison with AWS and ML when used for routine nasotracheal intubation.

## Methods

### Ethics approval and consent to participate

This study was approved by the Ethics Committee at the Aichi Gakuin University School of Dentistry (Approval No. 456) and was registered in advance as a clinical trial in the UMIN-CTR (Registration No. UMIN000023506). After providing an adequate explanation regarding the aims of the research to all subjects, we obtained written informed consent from all the patients.

### Subjects

**Fig. 1:**
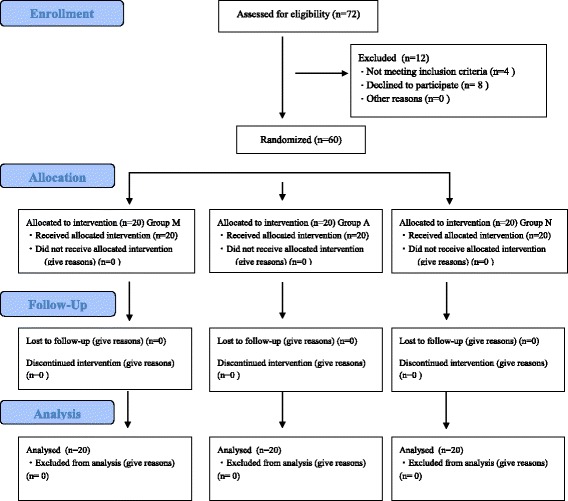
Consolidated Standards of Reporting Trials (CONSORT) recommended description of patient recruitment

Overall, 60 ASA-PS 1–2 patients between 20 and 70 years of age who were scheduled to undergo nasotracheal intubation under general anesthesia were included in this study. Patients were randomly assigned to McGrath MAC group (hereafter referred to as “M group”: 20 patients), AWS group (hereafter referred to as “A group”: 20 patients), and ML group (hereafter referred to as “N group”: 20 patients) (Fig. 1). Exclusion criteria were as follows: patients who did not provide written informed consent, patients with rhinostenosis on a preoperative computed tomography (CT) scan, and patients in whom intubation was expected to be difficult (Mallampati grade 3 or more, thyromental distance less than 4 cm, and interincisal distance less than 3 cm).

### Method of anesthesia

The same method of anesthesia was employed for all patients, and no premedication was administered. After a patient walked independently to the operating theater, the standard monitors were attached and a left intravenous route was secured. Anesthesia was induced using propofol (1–2 mg/kg), remifentanil (0.2 μg/kg/min.), and fentanyl (100 μg), with rocuronium (0.6 mg/kg) used as a neuromuscular blocking agent. Until the effect of the neuromuscular blocking agent became apparent by assessing Train Of Four, mask ventilation was implemented for all patients using 100% oxygen. While mask ventilation was being performed, tramazoline nasal drops were administered to the nasal cavity, and nasotracheal intubation was conducted after the effect of the muscle relaxant was observed. The intubation procedure was as follows. To ensure tracheal intubation technique, the procedure was performed by six doctors who were all certified by the Japanese Dental Society of Anesthesiology and had more than 6 years of experience each. A thin type of Intlock without any modifications was used for nasotracheal intubation with AWS to enable the use of Magill forceps, if necessary. As for McG, a disposable blade, MAC4, was used. The nasotracheal intubation tube used in this study was a North Pro File™ Soft-Seal (Smith Medical, Tokyo), and the tube size used was 7.0 for males and 6.5 for females. Patients in A and M groups were intubated in a neutral position and patients in N group were intubated in the sniffing position. Measures such as adjusting the position of the head and applying cricoid pressure were performed as necessary. Magill forceps were also used as required during intubation. In this study, after verifying the absence of rhinostenosis at the time of the preoperative examination and the preoperative CT scan, nasotracheal intubation was performed through the right nasal cavity in all patients [12].

### Evaluation parameters

The preoperative patient attributes of sex, age, height, and weight were evaluated. Preoperative airway evaluation items included Mallampati grade, thyromental distance, interincisal distance, mandibular movement, and neck movement. The primary outcome, were tracheal intubation time. Cormack Lehane grade during tracheal intubation (laryngoscopy was executed at the epiglottis valley in M and N groups, and executed underneath the epiglottis in A group), Magill forceps usage, head position adjustment requirement, and cricoid pressure application were secondly outcomes. The start time for tracheal intubation was deemed to be the time when the tracheal intubation tube passed through the nasal cavity and the patient’s mouth was opened with the cross finger maneuver. The finish time was deemed to be the time when the patient’s chest was seen to rise after tracheal intubation was completed. The tube was connected to the anesthesia circuit, and manual ventilation was initiated. Potential complications, if any, during nasotracheal intubation, included decreased SpO_2_ during the tracheal intubation procedure, bleeding from the oral mucosa, lip injuries, teeth damage, esophageal intubation, and postoperative pharyngeal pain.

### Statistical examination

We calculated the required minimum number of samples (*n* = 51 cases; M group, 17 cases; A group, 17 cases; N group, 17 cases; effect size, 0.57; α-error, 0.05; power, 0.95). The effect size was calculated on the basis of the statistical results of a pilot study wherein intubation time (the time difference between the 3 groups) was used as a standard [M group, 10 cases; A group, 10 cases; N group, 10 cases; analysis of variance (ANOVA), *p* < 0.01; Intergroup variation, 859.2; error variation, 654.9]. One-way ANOVA and multiple comparisons using the Tukey’s method were used for age, height, weight and tracheal intubation time. The chi-squared independence test m × n contingency table was used for sex, thyromental distance, inter incisor distance, ability to advance maxilla, cervical spine mobility, number of intubation attempts, and complications. The Kruskal–Wallis test and the Mann–Whitney test were used for Mallampati grade and Cormack Lehane grade. The level of statistical significance was set at *p* < 0.05.

## Results

From August to November 2016, 72 patients were selected as subjects for this study. Figure 1 shows the consort flow diagram, and the 60 subjects were randomly assigned into M group, A group, and N group. None of the subjects dropped out during the trial.

Table 1 shows patient sex, age, height, weight, and preoperative respiratory tract findings. No statistical differences in any parameters were observed among the three groups.

**Table 1:** Characterristics and airway assesment data of patients undergoing nasotracheal intubation

		Macintosh	McGrath	Airway scope	*P* value
Sex	Man	11	12	12	0,90 NS
	Woman	9	8	8	
Age: years		34.3 ± 12.2	36.3 ± 13.6	33.8 ± 13.4	0,93 NS
Hgigt: cm		162.1 ± 6.5	162.8 ± 8.1	167.4 ± 9.1	0,17 NS
Weight: kg		58.1 ± 10.3	55.4 ± 9.4	55.4 ± 9.7	0,60 NS
Mallampati: 1/2/3/4		8/12//0/0	9/11/0/0	10/10/0/0	0,86 NS
Thyromental Distance	< 4 cm ~ < 6 cm	15	14	15	0,91 NS
	≥ 6 cm	5	6	5	
Interincisal Distance	< 3 cm ~ <4 cm	6	5	5	0.91 NS
	≥ 4 cm	14	15	15	
Ability to advance maxxila	Yes	16	14	15	0,76 NS
	No	4	6	5	
Cervlacl supine mobility	Normal (> 90°)	19	19	18	0,76 NS
	Abnormal (< 90°)	1	1	2	

Values are mean ± SD or number. NS means Not significance

Tables 2 shows tracheal intubation information for the three groups. Tracheal intubation time was 26.8 ± 5.7 s (mean ± standard deviation) for M group, 36.4 ± 11.0 s for A group, and 36.5 ± 8.9 s for N group, indicating significantly shorter tracheal intubation time in M group than in A group (*p* < 0.01) and N group (*p* < 0.01). The Cormack Lehane grade was significantly improved in M group compared to N group (*p* < 0.05). In terms of the number of intubation attempts, no statistically significant difference was observed among the three groups regardless of whether Magill forceps were used or whether cricoid pressure was applied. In terms of whether the head position was adjusted, the head was tilted back more often during intubation for N group than for M and A groups (*p* < 0.01). With regard to desaturation, lip bleeding, and dental injury, there were no statistically significant difference among the three groups. Although there were no statistically significant difference, mucosal bleeding and oesophageal intubation were observed in A group (*p =* 0.12). Also postoperative pharyngeal pain was observed less frequently in M group than in A and N groups (*p =* 0.19).

**Table 2:** Intubation characteristics and performance of the video laryngoscopes and the conventional Macintosh laryngoscope

Tracheal intubation characteristics and performance of the video laryngoscopes and the conventional macintosh laryngoscope				
	Macintosh	McGrath	Airway scope	*P* value
Tracheal Intubationtime: s	36.5 ± 8.9^a^	26.8 ± 5.7	36.4 ± 11.0^b^	*P* < 0.01
Cormack Lehane Grage 1/2/3/4	9/9/2/0^a^	17/3/0/0	13/7/0/0	*P* < 0,05
Number of intubation attempts 1/2/3	18/2/0/0	19/1/0/0	17/2/1/0	0.64 NS
Manoeuvres to aid intubation				
Using magil forceps	5	0	4	0.06 NS
Readjust patient’s head	10	0	0	*P* < 0,01
External laryngeal pressure	3	3	4	0.88 NS
Complications				
Desaturation	0	0	0	N/A
Mucosal bleeding	0	0	2	0.12 NS
Lip bleeding	0	0	0	N/A
Dental Injury	0	0	0	N/A
Esophageal intubation	0	0	2	0.12 NS
Post operative sore throat	4	1	5	0.19 NS

Values are mean 1 SD or number. NS means Not significance. N/A means Not Applicable

^a^Macintosh vs McGrath

^b^McGrath vs Airway Scope

## Discussion

To observe the glottis using a laryngoscope, it is necessary to make a straight line from a visual point outside the oral cavity right through to the glottis. To achieve this, the head of the patient must be placed in an unnatural position at the time of tracheal intubation. Furthermore, when intubation is performed under direct vision using a laryngoscope, it is reportedly impossible to see the glottis clearly in 5–20% of patients [5]. When difficulties are encountered during laryngoscopy, application of excessive force to the laryngoscope may result in damage to the teeth and lips, which can cause an excessive increase in patient blood pressure and intracranial pressure. In addition, repeat intubation procedure using a laryngoscope may cause ventilation difficulties. Therefore, some doctors do not consider the use of a conventional ML to be ideal for tracheal intubation [5]. Video laryngoscopes were developed to address the aforementioned issues in recent years, and their popularity has increased as they enable tracheal intubation while verifying the position of the glottis on the monitor screen.

In this study, McGrath MAC offered an improved field of view during tracheal intubation compared with the conventional ML; in addition, the tracheal intubation time was reduced. The main reason for this reduction in tracheal intubation time when using McG appeared to be the high rate of glottis visibility. McG requires no special training even for doctors in the initial stages of practice, and it was reported that inexperienced doctors were able to recognize the glottis and achieve tracheal intubation with McG [1]. To ensure an accurate tracheal intubation technique, in this study, the tracheal intubation procedure was performed by six doctors who were certified by the Japanese Dental Society of Anesthesiology and had more than 6 years of experience each. The manner in which McG is used is nearly the same as that of ML, which has been the predominant tracheal intubation device used to date; therefore, the doctors who performed tracheal intubation in this study were well versed in the use of ML and stated that glottis visibility (rate) was high with McG. Because of the high rate of glottis visibility, there was no need to use Magill forceps or other procedures to insert the tube, and we believe that this factor contributed to the reduction of tracheal intubation time.

Meanwhile, compared with using only a video laryngoscope, tracheal intubation time using McG was shortened by approximately 12 s when compared with AWS. We believe that the position of the head and the ease of use for the video laryngoscope were contributing factors to the time reduction. With regard to the position of the head, according to a study reporting nasotracheal intubation on a mannequin assisted by the use of AWS [13], a dramatic reduction in tracheal intubation success rate was reported in the group in which the head was tilted forward compared to the group in which the head was tilted back. Although the direction in which the intubation tube moves during nasotracheal intubation significantly depends on the anatomic curvature of the area from the nasal cavity to the pharynx, it is suggested that the anatomic curvature is located closer to the dorsal side of the esophagus (the back) when the head is tilted forward than it is when the head is tilted back. In this study, of the patients in whom intubation was performed using AWS, 20 patients underwent intubation in a neutral position. By carrying out intubation in a neutral position, without the head tilted forward and without bending the anatomic curvature from the nasal cavity to the pharynx in the direction of the abdomen, we believe that the insertion of the intubation tube may have been impeded. The intubation time was shorter with McG, despite the fact that intubation was performed in a neutral position in all cases. This may have been not only because of the fact that images could be viewed indirectly on the monitor screen with McG but also because the tube could be quickly inserted in the direction of the glottis under direct vision, suggesting that the anatomic curvature from the nasal cavity to the pharynx did not have much of an influence. If tracheal intubation in the A group had been performed in all cases with the head tilted back, while the possibility remains that tube progress may have been impeded because of the tube hitting the front wall of the trachea, we believe that the intubation time may have been shortened. In terms of operability, AWS insertion method differs from that of the conventional MLs and is considered similar to the method used with laryngeal mask airways [5]. By inserting the blade with the head and neck in a natural position, the tip will naturally come to rest on the lower part of the epiglottis on the side of the glottis, and the use of this method requires some experience. In terms of operability, technicians familiar with the use of MLs on a day-to-day basis may find McG easier to use than AWS. If we focus on tube insertion, as previously mentioned, while the tube can be inserted into the glottis with McG either under direct vision or indirectly using the monitor, the tube can only be inserted indirectly using the monitor with AWS. In actual practice, despite the fact that the glottis is visible on the monitor screen with AWS, we experienced a number of cases where it was difficult to insert the tube. This may also have influenced the intubation time.

Although no statistical difference was observed for postoperative pharyngeal pain with McG compared with AWS and ML, the frequency of occurrence was lower with the former. As the oral cavity, pharynx, and larynx are all in a straight line, the shape of the blade used with video laryngoscopes imposes no non-physiological load. Therefore, the stress on the body is minimal. Thus, although video laryngoscopes may possibly reduce postoperative pharyngeal pain, such pain occurred at approximately the same frequency with AWS as it did with ML. With AWS, in cases where it was difficult to insert the tube using only the monitor screen, the area surrounding the glottis was hit several times by the tube despite the ability to easily recognize the glottis. This may contribute to the onset of postoperative pharyngeal pain when using AWS.

This study had some limitations. First, although the tracheal intubation procedure was performed by six experienced doctors, there may have been some differences in terms of the technique used. If the procedure had been performed by the same doctor in all cases, the results may have been different. Second, Pentax AWS video laryngoscope with its channeled blade was designated primarily for orotracheal intubation although some reports describing nasotracheal intubation have already been published. Technically, a bulky channeled blade of the Pentax AWS may worsen intubation conditions during nasotracheal intubation, mainly manipulation with the Magill forceps.

Third, although there are no statistical difference between McG and AWS in the visibility (Cormack Lehane grade), the visibility for the AWS is somewhere in conflict with other literature [14–16].We do not use AWS on a daily basis, and there is a possibility that familiarity has influenced the results. Furthermore, although the usefulness of McG at the time of nasotracheal intubation was demonstrated in this study, if adequate mask ventilation is performed using 100% oxygen prior to tracheal intubation, there is said to be a leeway of approximately 7–8 min in a healthy adults until SpO_2_ drops below 90% and the patient enters a hypoxic state after breathing has stopped [17]. Therefore, in clinical practice, the reduction of intubation time by approximately 10 s may have limited significance.

## Conclusions

This study addressed the usefulness of the McGrath MAC laryngoscope in comparison with Airwayscope and Macintosh laryngoscope when used for routine nasotracheal intubation. McGrath MAC improved the field of view during intubation and shortened the intubation time compared with Airway scope and conventional laryngoscopes, thus making nasotracheal intubation easier to carry out.

## Abbreviations

A group: AWS group
AWS: Airway scope
M group: McG group
McG: McGrath® video laryngoscope
ML: Macintosh laryngoscope
N group: ML group
NTI: Nasotracheal intubation
